# Ultrasound-Assisted Deep Eutectic Solvent Three-Phase Partitioning System for Extraction of Polysaccharides from Longan Shell: Process Optimization, Physicochemical Properties, Structural Characterization, and Antioxidant Activities

**DOI:** 10.3390/foods15112041

**Published:** 2026-06-05

**Authors:** Xinyu Zhang, Pengkun Xu, Jing Yao, Junhong Hou, Yutong Xu, Hao Chen

**Affiliations:** 1 Marine College, Shandong University, Weihai 264209, China; 202300810059@mail.sdu.edu.cn (X.Z.); 202300810034@mail.sdu.edu.cn (P.X.); 17785346253@163.com (J.Y.); 202500810054@mail.sdu.edu.cn (J.H.); 202500810215@mail.sdu.edu.cn (Y.X.); 2 Shandong Key Laboratory of Intelligent Marine Engineering Geology, Environment and Equipment, Qingdao 266237, China

**Keywords:** three-phase partitioning, deep eutectic solvents, longan shell polysaccharide, antioxidant activity, recyclable extractant, response surface methodology

## Abstract

In this study, a methodology that combines ultrasound-enhanced extraction with the use of hydrophobic deep eutectic solvents (DESs) and three-phase partitioning (TPP) was presented for the green isolation of polysaccharides from longan shells (LSP). The extraction system was a DES composed of an equal molar ratio of dodecanoic acid and octanoic acid, which was used as the separation medium. Generally, the main phase separation mechanisms involved in the purification of the polysaccharide were investigated. The ideal operational parameters were found through systematic optimization by the single-variable experiment with the response surface methodology, i.e., the extraction temperature of 63.8 °C, the phase volume ratio of 1:1.04 (*v*/*v*), and the ammonium sulfate concentration of 26.3%. The extraction efficiency is 2.42 ± 0.03% for LSP when the above operational parameters are used. The structural characterization showed that the isolated LSP is an acidic heteropolysaccharide rich in galacturonic acid and arabinose. It was also shown that the molecular architecture of LSP includes both types of glycosidic bonds, which are also of importance for its physicochemical properties. The polysaccharide exhibits an open fibrous network structure. Notably, the DES maintained stable performance over five successive reuses without significant degradation. Concerning the antioxidant capacity, LSP at 0.4 mg/mL showed 96.6 ± 2.0% inhibition of ABTS radical, and showed an iron-reducing capacity of 68.67 ± 2.02 micromol Trolox per gram (concentration-dependent effect). These results are present a new method for the sustainable extraction of bioactive macromolecules.

## 1. Introduction

The longan (*Dimocarpus longan Lour.*) is an evergreen species that belongs to the genus *Dimocarpus* of the *Sapindaceae* family and grows in tropical and subtropical regions. The edible pulp and usually discarded pericarp of this fruit have a large amount of bioactive components, such as polysaccharides, polyphenols, and some essential micronutrients. It has been proven that these phytochemicals are involved in many different biological regulatory mechanisms, such as scavenging free radicals, modulating inflammatory responses and maintaining metabolic homeostasis [1,2]. In general, the polysaccharides extracted from the longan shell also have versatile biological activities, such as antioxidant effects, antimicrobial properties and glycemic control, which indicates great potential for their practical utilization [3]. At present, the industrial processing of longan only involves the manufacture of the dried pulp (usually called longan aril).

The main commercial products are the edible portion of longan (mainly the flesh) and its canned goods. Most of the waste from the shells is either wasted or used as inferior fuel; there is a large amount of resource waste and ecological pollution. Some previous investigations have considered the use of longan shells for the production of crude fiber or activated charcoal [4]. However, it has not been explored whether the shells are able to produce some beneficial components, such as polyphenols and polysaccharides. Therefore, it is necessary to establish efficient techniques for extracting and purifying polysaccharides from these shells to increase the economic value of this agricultural waste material.

The choice of the extraction techniques influences both the amount and the structure of the naturally derived polysaccharides, and, therefore, the possible uses. Although traditional hot water extraction (HWE) is still widely used, because of the simplicity of implementation, the economical advantages and the safety considerations, this method has several limits: prolonged processing time, relatively low extraction efficiency, poor selectivity of the target polysaccharide, and possible heat-induced damage that could reduce the biological activity [5]. In order to solve these problems, some modern enhanced extraction approaches were proposed, such as ultrasound-facilitated, microwave-supported freeze–thaw repetition, and enzyme-based techniques [6,7]. One of the most interesting is deep eutectic solvents (DESs), which forms an eco-friendly solvent system obtained by hydrogen bonding between donor and acceptor molecules that has several outstanding features such as low evaporation rates, improved extraction yields, high recyclability, and excellent dissolving power [8]. They have been shown to be very efficient solvents to dissolve polysaccharides, proteins, polyphenolic compounds, flavonoid derivatives, and alkaloid molecules of different bioactive substances, which can also be isolated effectively while retaining their biological activity [9]. Since the DES at room temperature is in the liquid state because of the depressed melting point, it enables close contact with the target substances, and as a consequence the extraction effectiveness is increased.

Three-phase partitioning (TPP) is a simple, cost-effective, and green purification method. The technique works by adding some inorganic salts and some organic solvents to the raw extracts. This gives the formation of a three-layer system: a top layer with small-molecule pigments and fats, a middle layer of precipitated proteins, and a bottom phase that is abundant in hydrophilic compounds, such as polysaccharides, which allows straightforward polysaccharide extraction [10]. TPP was shown to be very adaptable to large-scale applications in mild conditions. However, the commonly used solvent, tert-butanol, poses significant safety risks due to its high volatility, flammability, and explosion hazard, as well as its toxicity [11]. There is an urgent need to find a green and safe alternative solvent. DES extraction and TPP have been reported in polysaccharide research. More importantly, the safety of tert-butanol in the existing TPP system limits its application in the field of food and medicine, and the designability and low toxicity of DES provide the possibility to replace tert-butanol [12].

Among various emerging techniques, ultrasound-assisted extraction offers practical advantages over microwave- or enzyme-assisted methods for the DES-TPP system. Unlike microwave irradiation, which generates localized hotspots and causes thermal degradation of polysaccharides [7], ultrasound relies on acoustic cavitation to disrupt cell walls under mild bulk temperatures [13]. Compared with enzyme-assisted extraction, ultrasound requires no costly enzymes or strict pH control [6], making it more robust and scalable. Therefore, ultrasound was selected as the intensification technique for DES-TPP in this work. Although deep eutectic solvents have been widely used for polysaccharide extraction [8], and three-phase partitioning has been applied for polysaccharide purification using tert-butanol [14], the integration of hydrophobic DES into a TPP system for simultaneous extraction and purification of polysaccharides from longan shells has not been previously reported. Compared with conventional DES-based solid–liquid extraction, this approach enables one-step selective purification without additional precipitation steps. Compared with tert-butanol TPP, our method replaces a toxic and volatile solvent with a non-toxic, recyclable DES.

In this work, we set up an ultrasound-assisted deep eutectic solvent three-phase partitioning (UA-DES-TPP) method for extracting polysaccharides from longan shells. The main advantages—higher yield, recyclable DES, mild conditions—were examined, and the LSP extracted from TPP (LSP-TPP) was compared with hot water-extracted LSP (LSP-HWE) in terms of its structure, physicochemical properties, and antioxidant activity. To the best of our knowledge, this is the first time UA-DES-TPP has been applied to LSP, and also the first to systematically compare product quality.

## 2. Materials and Methods

### 2.1. Materials and Reagents

The longan shells used in this work were from Guangxi Province. The chemical compounds were from the following commercial sources: the petroleum ether, the absolute ethanol, and the lauric acid (hydrogen bond acceptors, HBAs) were from Xilong Scientific Co., Ltd., Guangdong Province, China. The hydrogen bond donors (HBDs), pelargonic acid, capric acid, and caprylic acid, as well as the ammonium sulfate (99% purity, used to make the three-phase system), were from Tianjin Kemiou Chemical Reagent Company, Tianjin, China. The ten standard monosaccharides used in the chromatographic analysis of this work were ribose, arabinose, rhamnose, xylose, glucuronic acid, glucose, galactose, galacturonic acid, mannose and fucose. The others were acetonitrile (as the HPLC eluent), bovine serum albumin (BSA, used for protein quantification with Coomassie brilliant blue method), D-glucose, and phenol (used in the phenol–sulfuric acid procedure). The reagents used for polysaccharide measurement were purchased from Shanghai McLin Biochemical Technology Co., Ltd., Shanghai, China. The reagents of chloroform (used in monosaccharide extraction after the derivatization) and PMP were used. The derivatization agent 1-phenyl-3-methyl-5-pyrazolone for monosaccharide analysis was purchased from Shanghai Aladdin Biochemical Technology Co., Ltd., Shanghai, China. Several compounds used for the evaluation of antioxidant properties were bought from Fuzhou Feijing Biotechnology Co., Ltd., Fujian Province, China: ABTS (2,2′-azino-bis-3-ethylbenzothiazoline-6-sulfonic acid), Trolox (a water-soluble vitamin E derivative, used as a reference standard), and FRAP reagent (containing TPTZ, ferric ions, and acetate buffer solution). Finally, the mobile phase constituent for HPLC analysis of the sodium dihydrogen phosphate buffer was bought from Hangzhou Xinran Biotechnology Co., Ltd., Zhejiang Province, China.

### 2.2. Preprocessing of Longan Shell

The longan shell was dried and ground into particles of a fine size by means of a mechanical method, and the powder was sieved by a 60-mesh sieve. The powder was put in the Soxhlet extractor with the petroleum ether to remove the lipids with the proportion of solvent to the material at 10:1. The mixture was extracted for two hours at 40 °C in a water bath system with very strict control of the temperature [15]. After removing the lipids, the processed powder was put in a ventilated drying oven (Model DZF-6020MBE, Boxun, Shanghai, China) at 30 °C for twelve hours to let the solvent evaporate well. In the bleaching stage, the defatted powder was put into a new Soxhlet apparatus with an 80 percent ethanol solution with the proportion of the solvent to the solid at 20:1, and it was heated at 40 °C for 150 min. After the pigment extraction was done, the treated material was put into the temperature-regulated dryer for another twelve hours of drying. The drying was done at 30 °C with ventilation to remove the residual ethanol. The final dried powder product was collected and stored in temperature-regulated conditions.

### 2.3. Three-Phase Partitioning Method Enhanced by Ultrasound for LSP Isolation

The isolation of LSP was done by UA-TPP. Conventional TPP using tert-butanol was performed as a positive control to evaluate the relative performance of the DES-TPP system. Despite its known safety concerns, small-scale use (20 mL) under standard laboratory safety precautions (fume hood, PPE) was considered acceptable for this controlled comparison. The extraction method followed a previously reported procedure with minor modifications [16]. Briefly, 1 g of powdered longan shell material was soaked in the 25% ammonium sulfate aqueous solution. The material to solvent ratio was set as 1:20. We added 20 mL of tert-butanol and did the extraction under ultrasonic conditions (300 W power) at 55 °C for 25 min. After the extraction, the mixture was centrifuged at 4000 rpm for 10 min to separate the lower aqueous phase. We put the aqueous fraction in a 35 kDa molecular weight cutoff dialysis membrane and dialyzed continuously against distilled water for 2 days. After the dialysis, we lyophilized the solution to get a polysaccharide specimen. The LSP yield determination was done by the phenol–sulfuric acid method using glucose as the calibration standard.

### 2.4. Ultrasound-Assisted Deep Eutectic Solvent-Based Three-Phase Partitioning for LSP Extraction

#### 2.4.1. Preparation of Deep Eutectic Solvent Mixtures

The customized solvent system designed for the isolation of polysaccharides was prepared as follows: the measured amount of hydrogen bond acceptor and hydrogen bond donor substances were mixed in the round-bottom vessel. The combination was kept under constant stirring at 80 °C until full dissolution and visual transparency [17]. The physical and chemical properties of the formulated DES variants are illustrated in Table 1.

#### 2.4.2. Optimization of DES Solvent for LSP Extraction

According to the method of Dong G et al., the experiment was optimized [18]. A total of 1 g longan shell powder was mixed with different DESs (DES-1, DES-2, DES-3) at a solid–liquid ratio of 1:20, and 20 mL 20 wt% ammonium sulfate solution was added to form a TPP system. The extraction temperature was 55 °C, the ultrasonic power was 300 W, and the ultrasonic time was 25 min. After extraction, centrifuge at 4000 r/min for 10 min, and collect the lower layer. The lower components were poured into a 35 kDa retention dialysis bag and dialyzed with flowing deionized water for 48 h, and the dialysate was freeze-dried to obtain a polysaccharide sample. The polysaccharides extracted with DES-1~DES-3 were labeled as LSP-DES-1~LSP-DES-3, respectively. The yield of LSP-DESs was determined by the phenol–sulfuric acid method and glucose standard curve.

#### 2.4.3. Single-Factor Experiments of LSP Extraction

If the fixed DES and the same ultrasonic conditions are used, several critical elements are involved in the performance of UA-DES-TPP extraction. They are the following: the processing time, the working temperature, the ammonium sulfate content, the ratio between the liquid and solid, and the volume ratio between the upper and the lower phase. In order to find the optimal conditions for the ultrasound-assisted DES extraction of the LSP, without changing the solvent choice or the acoustic parameters, a sequence of individual factor analysis was done. All these tests showed how the different time of extraction (from 15 to 55 min), the working temperature (from 35 to 75 °C), the ammonium sulfate concentration (from 10 to 30% *v*/*v*), the ratio between the liquid and the solid (from 1:10 to 1:30), and the volume proportion between the upper and the lower phase (0.5:1–2.5:1) influenced the yield of the LSP obtained by the method of UA-DES-TPP. In the methodology of the research, one variable was modified at a time, and the rest of the parameters were kept fixed at the central values. For example, when the effect of the extraction time is studied, the experiments are carried out at five different times (15, 25, 35, 45, and 55 min), and the other conditions, for example, the temperature or the concentration of the ammonium sulfate, are kept fixed in the central condition. This gives the opportunity to study the effect of each variable on the extraction efficiency separately. In the course of the investigation, the following experimental conditions were kept constant: temperature 55 °C, solid concentration 20%, liquid/solid ratio 1:20, and phase volume ratio upper/lower layers 1.5:1. All these parameters were controlled and kept the same for the whole of the study.

### 2.5. Response Surface Optimization of LSP Extraction

In order to increase the efficiency of the extraction, response surface methodology (RSM) with a second-order polynomial model was carried out. From the five factors examined in the single-factor experiments (ultrasonic time, solid–liquid ratio, ammonium sulfate concentration, phase volume ratio, and extraction temperature), three parameters—extraction temperature, ammonium sulfate concentration, and phase volume ratio—showed pronounced effects on LSP yield, whereas ultrasonic time and solid–liquid ratio had relatively minor impacts under the tested conditions. Therefore, the three significant factors were selected for RSM optimization, while ultrasonic time and solid–liquid ratio were fixed at 25 min and 1:20 g/mL, respectively. The three selected factors to be studied were processing temperature (A), ammonium sulfate content (B) and solvent ratio (C). These elements are the basis of the Box–Behnken design (BBD), in which every variable was studied in three different intensities (−1, 0, +1). The research methodology consists of 17 different experimental arrangements, taking as the main criterion of assessment the LSP yield, as shown in Table 2. The mathematical model predicted ideal extraction parameters, which were then tested in practice to verify the reliability of the established predictive model.

The extraction conditions were optimized by the response surface methodology combined with the second-order polynomial model. The preliminary single-variable experiments defined the three factors which are supposed to have an influence on the process: the operating temperature (A), the (NH_4_)_2_SO_4_ content (B), and the solvent ratio (C). We chose these parameters as the main variables of the Box–Behnken design. In the experimental frame, these parameters are varied systematically by three fixed levels (−1, 0, +1), as shown in Table 2. After rigorous statistical evaluation, the optimal extraction conditions were identified and subsequently validated by laboratory trials to confirm the high predictability of the proposed mathematical model [19].

### 2.6. Yield and Purity

#### 2.6.1. Standard Curve Establishment

The reference curve was built by using the phenol–sulfate method with the glucose reference solutions (0–100 μg/mL). The optical density was read at 490 nm of wavelength. The linear relationship was written as follows:

(1) $$y\;=\;0.0140x\;-\;0.0117\;(R^{2}\;=\;0.9983)$$

In this formula, y is the measured light absorption, and x is the concentration of glucose in milligrams per milliliter.

For protein concentrations, we used the Bradford method with bovine serum albumin as a reference (0–100 mg/mL). Absorbance was measured at 595 nm. The relation of the line is as follows:

(2) $$y\;=\;0.0062x\;+\;0.0189\;(R^{2}\;=\;0.9994)$$

Here, y is the observed absorbance value, and x is the protein concentration in milligrams per milliliter.

#### 2.6.2. Polysaccharide Yield and Purity

The total carbohydrate content was determined by the phenol–sulfuric acid method using D-glucose as a standard. The protein was quantified by the Coomassie brilliant blue assay, using the calibration curve made with bovine serum albumin to determine the polysaccharide purity [20]. The extraction yield of LSP was given by the following equation:

LSP yield (%) = (LSP mass (g)/longan shell powder mass (g)) × 100%(3)

#### 2.6.3. UV-Vis Spectral Analysis

The quality of the LSP sample obtained via DES extraction was assessed with a T6 UV-Vis spectrophotometer (Beijing Spectrum Technology Co., Ltd., Beijing, China). The samples were mixed with distilled water to make homogeneous mixtures. The mixtures were passed through 0.22 m membrane filters and ultrapure water was used as a reference. The detailed spectral examination was done with a wavelength range from 200 to 600 nm. The sample purity was estimated by characteristic absorption patterns, which we detected during the spectral scanning.

### 2.7. Recycling and Reusing Procedure of DESs

In order to check the use and the effectiveness of DES in terms of regeneration and reuse after the LSP extraction, an investigation into the solvent recycling was performed. Indeed, after every round of extraction, the upper DES phase is separated and collected, and is directly used in the following TPP extraction procedures without any purification. This recycling was done for five cycles. If the recycled DES retains its extraction efficiency for polysaccharides, this will demonstrate high solvent reusability, suggesting that its functional characteristics remain largely intact.

### 2.8. Structural Characterization Assays

#### 2.8.1. Scanning Electron Microscopy

The LSP samples were lyophilized before SEM analysis. The specimens were sputter-coated with a thin layer of gold before microscopic observation. The surface morphology characterization was carried out by a field emission scanning electron microscope (Nano SEM 450, FEI Ltd., USA) with an accelerating voltage of 10 kV.

#### 2.8.2. Relative Molecular Weight Distribution

The size distribution of LSP molecules was determined by gel permeation chromatography (GPC), following a very similar methodology to that described by Yang. We used Agilent 1260 Infinity II high-performance liquid chromatography with an Agilent 7162A detection module (Agilent Technologies, Santa Clara, CA, USA). As a stationary phase, we used a PL aquagel-OH MIXED-H column (Agilent Technologies, Santa Clara, CA, USA) (83,007.5 mm); both the column compartment and detector were 40 °C. As a mobile phase, we used a 0.1 mol/L sodium nitrate aqueous solution. In order to put it in the GPC column, we dissolved the LSP in the solvent at 1 mg/mL, and we filtered through 0.22 m membranes. We injected 10 L samples; the solvent flow was the same at 1 mL/min. The molecular weight calibration was done with polystyrene standards; all the obtained molecular weights are relative.

#### 2.8.3. Fourier Transform Infrared Spectrum

The LSP samples were mixed with dry potassium bromide in a ratio of 1:100 in weight, uniformly mixed (manually ground in order to be completely mixed), and then pressed in transparent films. The prepared specimens were studied with a Bruker TENSOR27 Fourier transform infrared spectrometer (Bremen, Germany), with a scan from 4000 to 400 cm^−1^, resolution 4 cm^−1^ [21].

#### 2.8.4. Monosaccharide Composition

The measurement of the individual sugar units was done by a chemical modification process with 1-phenyl-3-methyl-5-pyrazolone (PMP), followed by separation and detection with a high-performance liquid chromatography instrument (Shimadzu LC20AD, Japan) [22]. The method was as follows: A set of ten different sugar reference compounds (mannose, ribose, rhamnose, glucuronic acid, galacturonic acid, glucose, galactose, xylose, arabinose, fucose) were weighed and mixed in purified water to make a composite standard solution where each had a concentration of 50 μg/mL. From the prepared solution, 250 μL is pipetted into a 5 mL centrifugation vessel. Then 250 μL of 0.6 M sodium hydroxide solution and 500 μL of 0.4 mol/L PMP dissolved in methanol is added to the aliquot. The solution is kept at 70 °C for one hour to make the chemical transformation, then the sample is immediately kept in an ice-water mixture for ten minutes.

For pH adjustment, 0.3 M HCl (500 μL) was added to the solution and chloroform (1 mL) was added. Both liquids were agitated with a vortex mixer at an intensity as intense as possible for one minute and then centrifuged at 3000 rpm for ten minutes. The aqueous phase was separated carefully and this extraction was done three times in all.

An aliquot of the weighed sample was put in a 10 mL volumetric flask. A total of 3.0 mL of 2 M trifluoroacetic acid was put in a 10 mL ampoule and the nitrogen gas was displaced and the ampoule was sealed hermetically. Acid-catalyzed hydrolysis was run at 120 °C for ten hours. After cooling, 1.0 mL of the hydrolyzed product was added to methanol, and the material was dried under nitrogen flow in order to remove the traces of TFA. The desiccated material was dissolved in 1 mL of deionized water.

A precise volume of 250 μL was taken from the prepared solution and transferred to a 5 mL microcentrifuge tube. Then 250 μL of 0.6 M sodium hydroxide and 500 μL of 0.4 M PMP–methanol solution were added to the tube. The mixture was kept at 70 °C for 60 min for reaction and then immediately kept in an ice-water mixture for 10 min. Afterwards, 500 μL of 0.3 M hydrochloric acid was added to the solution to neutralize it. The mixture was taken for liquid–liquid extraction with 1 mL of chloroform with the use of vigorous vortex mixing for 60 s. Then it was centrifuged at 3000 rpm for 10 min and the upper layer was collected. This extraction was repeated twice more. The combined organic phases were analyzed with high-performance liquid chromatography. The chromatographic parameters were: column: Xtimate C18 (4.6 × 200 mm, 5 μm); detector: LC20AD. The eluent was 83:17 of 0.05 M potassium dihydrogen phosphate buffer (pH adjusted to 6.70 with sodium hydroxide) and acetonitrile. The analysis was conducted at the detection wavelength of 250 nm with a constant solvent flow rate. The flow rate was 1.0 mL per minute. The injection volume was fixed to 20 μL and the column was kept at 30 °C.

### 2.9. Analysis of Physical and Chemical Properties

#### 2.9.1. Determination of pH Value

We prepared an aqueous solution containing 0.5% (weight per volume) LSP in distilled water. The pH measurement was performed with a PHS-25 pH meter (Shanghai Lemagnetic Instrument Co., Ltd., Shanghai, China).

#### 2.9.2. Examination of Solubility Characteristics

The solubility properties of LSP were tested at 25 °C, 60 °C, and 80 °C. A weighed amount of LSP was put in distilled water in order to get a 0.5% *w*/*v* solution. The suspension was mixed by stirring in a temperature-controlled shaking water bath for 60 min at each test temperature. The solution was then centrifuged at 10,000 rpm for 10 min in order to remove the insoluble polysaccharide residues. The transparent liquid phase was pipetted into previously weighed microcentrifuge tubes. After freeze drying to constant weight, the dissolved polysaccharide concentration in the supernatant was measured to calculate both solubility and dissolution rate at each temperature. The solubility was quantitatively assessed with the following mathematical formula:

(4) $$\mathrm{Solubility}(\%)\;=\;\frac{W_{2}\;-\;W_{0}}{W_{1}}\;\times \;100$$

where W_0_ represents the weight of the empty tube, W_1_ denotes the initial sample weight, and W_2_ indicates the sample weight post-lyophilization.

#### 2.9.3. Measurement of Zeta Potential

A specific amount of LSP was weighed and dissolved in distilled water to make 0.05% (*w*/*v*) suspension of LSP. The pH was adjusted to 3.0, 5.0, 7.0, 9.0 and 11.0 with 0.1 M HCl and 0.1 M NaOH solutions, respectively. A total of 10 mmol/L NaCl was added to ensure all the samples had the same ionic strength. The solutions were filtered through 0.45 μm membranes before the test. The surface charge properties of LSP were obtained at 25 °C from the zeta potential using a ZEN3600 nanoparticle analyzer (Malvern Panalytical Ltd., Malvern, UK).

#### 2.9.4. Apparent Color Analysis

The color characteristics of the polysaccharide solution were analyzed with a relatively sophisticated color measurement instrument (NR 110, Shenzhen Sanenchi Technology Co., Ltd., Shenzhen, China), and the chromatic properties were numerically evaluated with CIE Lab color space [23]. The analysis is in the frame with three chief variables: L—level of brightness; a—the chromatic scale between red and green (positive figures are the red, negative values are the green); and b—the color scale from yellow to blue (positive numbers are the yellow, negative values are the blue). For the experiments, the LSP samples were prepared in water-based solvents at a 0.1% mass-to-volume ratio, while the distilled water was used as the control. The chromatic coordinates L*, a*, and b* were recorded for every sample solution, and then the total color difference metric ΔE was calculated.

(5) $$\Delta E=\sqrt{{(L\;-\;L_{1})}^{2}+{(a\;-\;a_{1})}^{2}+{(b\;-\;b_{1})}^{2}}$$

In the computational formula, L, a and b are the colorimetric measurements of LSP and L_1_, while a_1_ and b_1_ are the corresponding colors of the reference water sample.

### 2.10. Determination of Functional Properties

#### 2.10.1. Thermal Stability

The thermal behavior of LSP specimens was examined using a thermogravimetric analysis instrument (TGA 2 SF/1100, METTLER TOLEDO, Greifensee, Switzerland). Under nitrogen gas flow conditions (50 mL/min), the temperature was increased at a constant rate of 10 °C per minute from 30 °C to 600 °C. Thermogravimetric analysis data provided information about mass reduction percentages, while peak degradation temperatures were identified through derivative thermogravimetric analysis.

#### 2.10.2. Antioxidant Capacity Assessment

The antioxidant properties of the LSP were evaluated following the methodology described by Amal D. Premarathna et al. [21], with the use of two different measures: ABTS free radical neutralization test and FRAP analysis [24]. In the first part of the preparation, 100 mg of the LSP powder was mixed with 10 mL of distilled water. This mixture was stirred constantly in light-protected conditions for 120 min to obtain the mother solution. From this solution, different test solutions were prepared by serial dilution to get final concentrations between 0.025 and 0.4 mg/mL.

The experimental procedure for the ABTS test was the same as below. In total, 20 μL of the sample being analyzed was combined with 180 μL of ABTS solution. The reaction mixture was incubated for 6 min in dark conditions at room temperature. After incubation, the absorbance of the solution was measured at 734 nm and was recorded as A_1_. In the control experimental solution, the sample was replaced with 20 μL of PBS buffer to measure the absorbance A_2_, and the ABTS solution was replaced with 180 μL of PBS to determine the reference absorbance A0. The capacity to scavenge ABTS radicals was measured by using the following calculation formula:

(6) $$\mathrm{ABTS}\;\mathrm{scavenging}\;\mathrm{rate}\;\left(\%\right)\;=\;\left[1\;-\;\frac{\left(A_{1}\;-\;A_{0}\right)}{A_{2}}\right]\;\times \;100\%$$

The FRAP working solution was composed of glacial acetic acid buffer (pH 3.6), 10 mM TPTZ solution and 20 mM FeCl_3_ solution at a volume ratio of 10:1:1. The standard curve was drawn with Trolox as the standard. The LSP sample solution was mixed with the FRAP working solution and incubated at 37 °C in the dark. The absorbance was measured at a wavelength of 593 nm and recorded as A_α_. The deionized water was used instead of the sample solution as the blank control to measure the absorbance of A_β_. Finally, the antioxidant capacity of the sample was expressed as Trolox-equivalent antioxidant capacity (TEAC) according to the Trolox standard curve.

(7) $$\mathrm{FRAP}\;\mathrm{capability}\;\left(\mu \mathrm{mol}\;\mathrm{TE}/g\right)=\left[\frac{\left(A_{\alpha }\;-\;A_{\beta }\right)}{\mathrm{Slope}\;\mathrm{of}\;\mathrm{the}\;\mathrm{standard}\;\mathrm{curve}}\right]\;\times \;100\%$$

### 2.11. Statistical Analysis

All experimental procedures were performed in three independent replicates and the results are shown as mean ± standard deviation (SD). Statistical evaluation was done in Origin 2026 software. One-way ANOVA followed by Tukey’s post hoc test were used to determine statistical differences, with a significance threshold of *p* < 0.05. RSM modeling and analysis of variance (ANOVA) for the optimization were performed using Design-Expert version 13 software.

## 3. Results

### 3.1. Screening and Selection of DES for LSP Extraction

**Figure 1:**
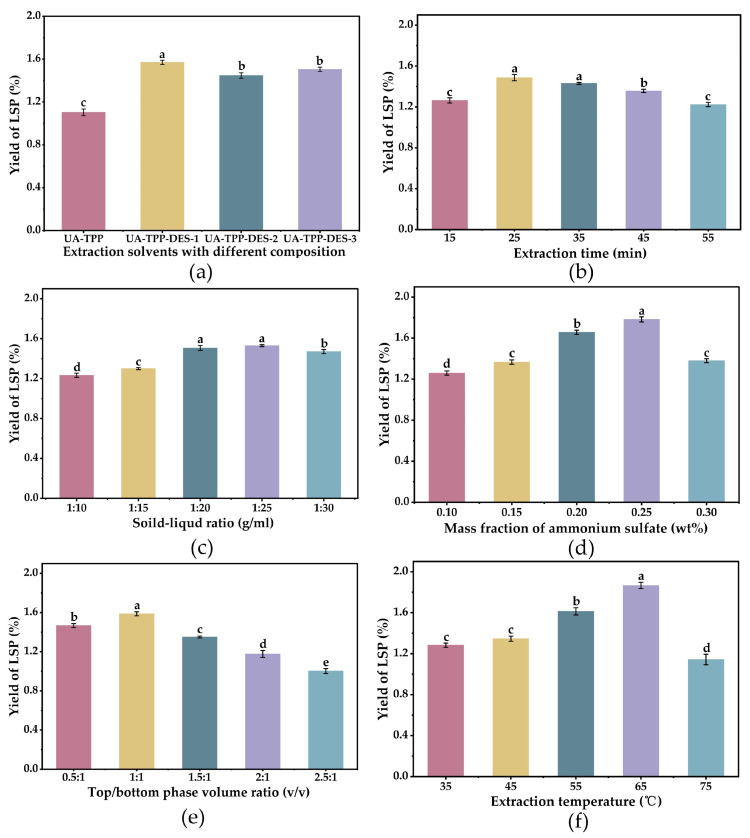
DES screening and selection and effects of single-factor parameters on the yield of LSP: (**a**) comparison of LSP yields using different DES systems and tert-butanol TPP; (**b**) ultrasonic time on LSP yield; (**c**) solid–liquid ratio on LSP yield; (**d**) ammonium sulfate concentration on LSP yield; (**e**) top/bottom phase volume ratio on LSP yield; (**f**) extraction temperature on LSP yield. The y-axis represents the LSP yield (%). The ultrasonic power was maintained at 300 W for all experiments. Different letters indicate statistical significance at *p* < 0.05, *n* = 3.

It was proven in several studies that the efficiency of the deep eutectic solvents for the isolation of the polysaccharides from the plant material is strongly dependent on the molecular composition of the DES. The change in the proportion of HBD to HBA changes basic solvent properties, for instance, the hydrogen bonding potential, the viscosity, and the whole of the polarity of the solvent, which then influence the solute–solvent interactions and the efficiency of the mass transfer [25]. In the case of the investigation of the best DES for the TPP-based LSP extraction, we investigated three different DES forms (DES-1, DES-2 and DES-3), while the tert-butanol TPP was used as a control. Figure 1a shows that the DES-TPP technique gives LSP yields from 1.45% to 1.57% that are much higher than the 1.10% yield of the tert-butanol TPP. These results show that the DES is better than the tert-butanol in TPP, and the benefit of the DES is mainly due to three reasons: first is the presence of carboxylic acid functional groups in the fatty acids. The formation of strong hydrogen bonding networks is facilitated.

The dissolution of polysaccharides is improved because of the interaction of the polysaccharides with DES [26]. Also, DES exhibits negligible volatility and high thermal stability, allowing it to maintain the three-phase boundary under ultrasonic conditions. In the case of DES-1, at the temperatures between 55 and 65 °C, we have lower viscosity which favors mass transfer by cavitation, and the tert-butanol tends to form the emulsions. In addition, DES-1, which is a 1:1 mixture of dodecanoic acid and octanoic acid, gives the highest yield of 1.57%. This is because of its C8/C12 fatty acid structure that is balanced between the hydrophilic and the hydrophobic characteristics, and the variation in the chain lengths helps to lower the viscosity. Subsequently, the TPP system (UA-TPP-DES-1) with DES-1 was selected for later experimental studies.

### 3.2. Single-Factor Experimental Analysis

A more complete and detailed investigation into the effect of all the factors on the production efficiency of the LSP was performed using the single-variable experimental methodology. The following parameters were considered: concentration of the ammonium sulfate, mass-to-volume ratio, ultrasound-assisted extraction time, processing temperature and distribution of the volume of the phases. All the experimental conditions were done in triplicate and the results are shown in Figure 1.

The extraction time using the technology of the ultrasound effect also influences the yield of LSP. If the treatment time was increased from 15 min to 25–35 min, then the rate of recovery of the polysaccharide is increased from 1.26% to 1.52%. This increase can be explained by the mechanical effects that are produced by the cavitation. Namely, the asymmetric collapse of microscopic bubbles in the vicinity of the solid–liquid boundaries produces powerful microstreams and local pressure waves. All of these physical forces together break the structures of the plant cells, allowing the release of intracellular polysaccharides and their motion in the extraction medium [13]. However, if processing time is more than 35 min, then the extraction efficiency decreases gradually and, for example, after 55 min, it is equal to 1.22%. These results show that about 25 min is the optimal extraction time for this method of application. The length of ultrasound treatment may cause polysaccharide molecular structures to be broken down by the excessive mechanical shear forces [27]. In the single variable analysis of the experimental conditions, all of the extraction parameters were kept the same, except for the duration of the extraction. In the following experiments, the same ultrasonic conditions (300 W, 25 min of processing) were used. After a comprehensive assessment of extraction yield and molecular integrity, we identified this particular time as the most suitable. This length of time is a compromise, because it is the same time as the maximum polysaccharide extraction, and it does not risk the molecule degradation that could occur if they were treated by ultrasound for too long.

When we examined the different solid-to-liquid proportions between 1:10 and 1:30, the recovery rate of LSP was a bell-shaped curve, and reached 1.53% when using 1:25. At first, in the case of changing the ratio from 1:10 (1.24%) to 1:20 (1.53%), the extraction of polysaccharide was improved, which may be attributed to the increasing volume of solvent, which can enhance the movement of molecules and dissolution [28,29]. In analytical comparison, we found no important difference in the output of 1:20 and 1:25 ratios (*p* > 0.05). However, when we increase the ratio to 1:30, the extraction performance declines obviously. After thorough evaluation of the effectiveness of extraction, the cost of solvent, and the need for subsequent processing, we decided the proportion of 1:20 is the best option. This decision reached an equilibrium between the polysaccharide yield and the reduction in solvent consumption while meeting the concentration specifications, which is in accordance with an extraction method that is eco-friendly and production that is sustainable.

For all the tested ammonium sulfate concentrations in the interval of 10% to 30%, the LSP extraction yield is a bell-shaped response curve with the highest productivity (1.78 ± 0.03%) for the 25% concentration. This probably reflects the complex interplay of salting-out phenomena and the regulation of the ionic strength of the solution. At an intermediate concentration of salt (under 25%), the electrolyte promotes selective precipitation by means of the charge neutralization effects of the contaminant proteins. The preferential removal of the contaminant proteins allows the previously bound polysaccharides, whether by means of electrostatic interactions or by means of the physical encapsulation of the polysaccharides in the matrix of the proteins, to be more easily transferred into the aqueous phase and so to increase the detectable extraction yields. At concentrations higher than 25%, a higher ionic strength reduces the surface tension differential between the DES and the aqueous phase, so that the phase partitioning is less distinct. A notable decrease in polysaccharide extraction efficiency was observed, with a diminished recovery, as reported in [30]. In fact, if we do the same experiments, repeated at the 25% concentration level, the same production trends are reproduced. The extraction yield was kept between 1.76% and 1.81%, which is a big change from the 1.36% to 1.40% that we recorded at the 30% concentration. These big changes also prove that the critical phase transition process was mainly affected by the charge screening effect.

The volume ratio of the two liquid layers affected the extraction yield of LSP. If the two phases retained the same volume (1:1 ratio), the highest polysaccharide recovery rate was 1.59 ± 0.02%. In this balanced configuration, mass transfer was promoted, the contact interface between phases was favorable, and the energy barrier at the boundary was reduced, so that the polysaccharide migration into the water-based layer was smoother. When the ratio was increased to 1.5:1, the extraction efficiency decreased, and the extraction output decreased to 1.35%. In this case, the volume of the deep eutectic solvent phase was widened and the chaotic fluid movements that are generated reduced the efficiency of the extraction, because the systematic polysaccharide transfer in the process is disturbed. It is an example of the negative effect of the flow disturbances on the effectiveness of the separation.

The extraction temperature also affected the production efficiency of LSP and the operation stability of the extraction. The peak of the extraction performance (1.87 ± 0.03%) was at 65 °C. For a temperature of 75 °C, the output decreased by 38.78% compared to the best extraction condition. The appropriate thermal conditions were able to improve the mobility of the molecules by decreasing the thickness of the solution and by favoring the solubility of the polysaccharide. For the temperatures above 65 °C, the hydrogen bond interactions among the DES constituents (caprylic acid–dodecanoic acid) are probably weakened; in this way, the three-phase extraction framework is destabilized and the effectiveness of the substance transfer decreases [31].

### 3.3. Process Optimization for TPP Extraction

#### 3.3.1. RSM Model Fitting

Preliminary investigations by single-factor testing found three variables that influenced, in a bigger or smaller way, the recovery rate of LSP: the conditions of heating the extraction, the mass percentage of ammonium sulfate in the solution and the volumetric proportion between phases. These important factors were optimized with response surface analysis, where the experimental design uses, as predictor variables, the extraction temperature (55–75 °C), the ammonium sulfate content (20–30% *v*/*v*) and the volumetric proportion between the phases (0.5:1 to 1.5:1) and the LSP recovery as the measured response. In the study, a Box–Behnken experimental arrangement for three variables is implemented, and 17 different trials are done, as in Table 3. Experimental details are in Table 4. The statistical processing of these results gave a quadratic regression equation that expresses the relation between LSP recovery and the examined parameters:

Y = 2.41 − 0.0365A + 0.1219B − 0.0431C − 0.0558AB + 0.1622AC + 0.0360BC − 0.2579A^2^ − 0.2347B^2^ − 0.3466C^2^ (8)

Y denotes the yield percentage of LSP, where A corresponds to the extraction temperature in degrees Celsius, B indicates the weight percentage of the ammonium sulfate solution concentration, and C signifies the volumetric ratio between the upper and lower phases.

Regarding the validity of the model, the regression results showed a strong statistical relevance (*p* < 0.001) and the lack-of-fit test did not show significant results (*p* = 0.3046 > 0.05), so we can say that the model is valid in the sense that it is able to predict the conditions of the maximum LSP yield. All the parameters are significantly affected in the yield of LSP: B, AC, A^2^, B^2^, and C^2^, in particular, show a strong effect; A, C, and AB show a strong, but not-as-strong, effect. If we analyze the F values present in Table 4, we can rank these factors by their function in the magnitude of the effect, so we have the order of importance: B > C > A.

The evaluation results presented in Section 3 demonstrate the strong effectiveness of the model. The coefficient of determination (R^2^ = 0.9923) and adjusted R^2^ (0.9826), with a difference of only 0.0098, indicate excellent model fit and reliable predictive accuracy. The small gap between these values, much less than 0.02, means the model calibration is sound. The low coefficient of variation (C.V.% = 1.94) confirms the consistency and dependability of the experimental procedure. After response surface analysis, the optimal extraction conditions are: temperature is set to 63.8 °C, phase volume ratio is kept at 1:1.04 (*v*/*v*), and the concentration of ammonium sulfate is fixed at 26.3 wt%. The projection extraction efficiency is 2.44%. It gives empirical evidence and theoretical support to the three-phase separation technique.

#### 3.3.2. Interactive Effects of Factors

**Figure 2:**
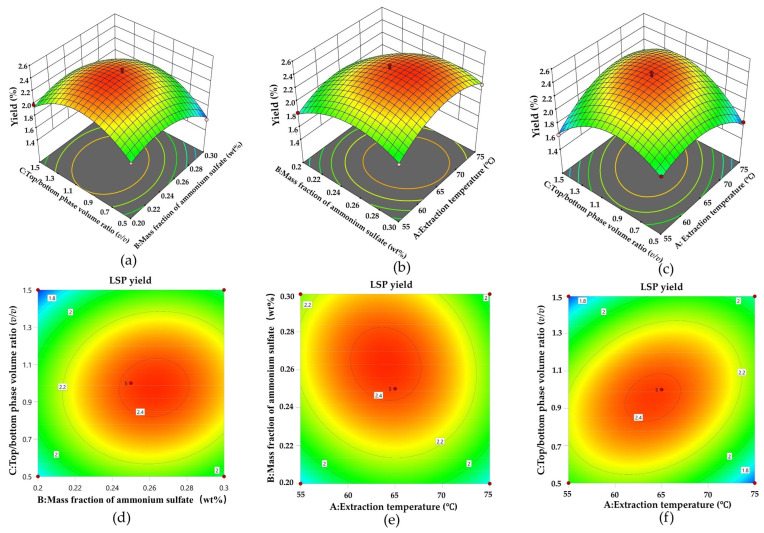
Three-dimensional response surface (**a**–**f**) showing the effect of interactions among three factors on the LSP yield. A is the extraction temperature (°C), B is the mass fraction of ammonium sulfate (wt%), and C is the top and bottom phase volume ratio (*v*/*v*).

The relationship between the interaction of various factors and LSP yield was further predicted by a 3D response surface, as shown in Figure 2a–c. The 3D response diagram composed of the extraction temperature (A), mass concentration of ammonium sulfate solution (B), and volume ratio of upper and lower phases (C) showed obvious nonlinear, steep characteristics, indicating that the interaction between the three factors had a decisive effect on the extraction results. Notably, the interaction between the extraction temperature (A) and the upper and lower phase volume ratio (C), AC, had a significant effect on the LSP yield. The positive interaction effect (coefficient + 0.1622) indicated that a suitable upper and lower phase ratio, and a certain temperature, could synergistically improve the extraction efficiency. At the same time, it should be noted that the interface mutation occurred when the temperature was greater than 70 °C, which reduced the yield. Through the projection map in Figure 2d–f and the mutation region of the response surface map, it can be observed that the upper and lower phase volume ratio (C) has a strong influence on the parabolic type (coefficient-0.431), which dominated the parabolic response results. The optimum extraction conditions predicted by the model were as follows: extraction temperature 63.8 °C, upper-to-lower phase volume ratio 1:1.04 *v*/*v*, ammonium sulfate solution concentration 26.3% wt%. Under these conditions, the predicted LSP yield was 2.43%. The actual yield obtained from the verification experiment was 2.42% ± 0.03% (*t*-test *p* = 0.118). The actual yield and theoretical yield were highly consistent, which fully proved the accuracy and reliability of the model.

### 3.4. Polysaccharide Yield and Purity Analysis

The extraction performance of LSP under the optimized conditions is shown in Table 5, in which the polysaccharide recovery rate, sugar content and protein composition are listed. In Table 5, the achieved extraction yield of 2.42% is very close to the standard LSP isolation results and the features of the TPP process. The actual yield (2.42%) deviated only slightly from the predicted value (2.44%). This little difference is still in the range of the standard experimental error. Therefore, it supports the use of this technique; UA-DES-TPP is practically applicable. Compared with the traditional hot water extraction methods (1.06%) reported in the past, the present method increases polysaccharide recovery by 128.30% [32]. Also, if we measure it against the ultrasonic-assisted extraction protocol (0.55%), the present method also increases the yield by 340% [33].

The analysis of the experimental data yielded a carbohydrate content of 89.03%, and the protein content, in this case, was still very low at 1.83%. This high degree of purity was obtained by the use of some specific separations. The phase separation mechanism, using DES and ammonium sulfate, set the protein exclusion zone and the selective extraction of a carbohydrate, leaving the proteins, as was described in [14]. Also, the use of a 35 kDa molecular weight cutoff membrane allowed the removal of the smaller molecular impurities. The special solvent characteristics of the caprylic acid–dodecanoic acid DES mixture were also important in this purification. This solvent system showed preferential solubility for the neutral polysaccharide and a poor capacity to dissolve proteins, which also improved the final polysaccharide purity.

**Figure 3:**
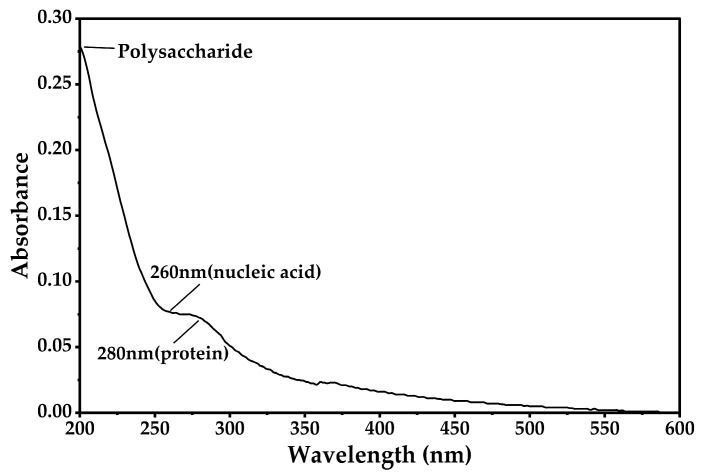
UV-Vis spectrum of LSP.

The analysis of the LSP purity was also supported by complete UV–visible spectroscopy in the full wavelength range, shown in Figure 3. The spectra showed typical end absorption at 200 nm, characteristic of polysaccharides, while no prominent absorption peaks were observed at 260 nm or 280 nm, suggesting low levels of nucleic acid and protein residues. This observation is consistent with the protein content determined by the Bradford assay (1.83%, Table 5). Taken together, these results support that the UA-DES-TPP method yields LSP with high purity.

### 3.5. Recycling and Reusing of DESs

**Figure 4:**
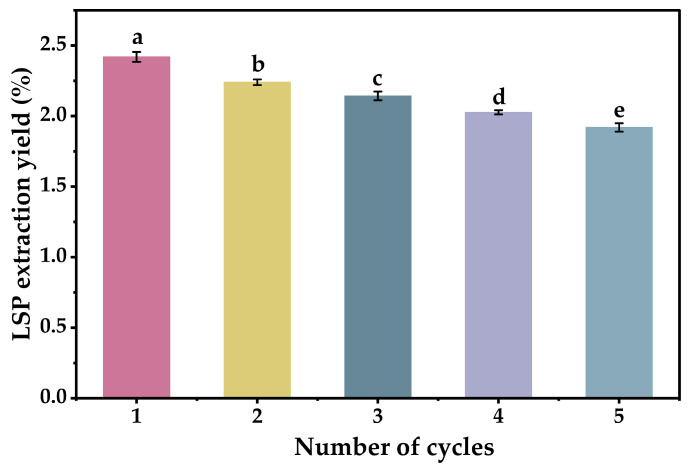
LSP extraction yield in recycling tests. Different letters indicate statistical significance at *p* < 0.05, *n* = 3.

The extraction performance of polysaccharides was used to test the durability and the recyclability of DESs. As is shown in Figure 4, after the first reuse, the yield was 2.24%, a decrease of only 0.18% compared to the initial extraction. In subsequent cycles, a slight decrease in polysaccharide yield was observed per cycle. But after five consecutive cycles, the extraction capacity was stable at more than 1.92%, which shows excellent reusability and the same performance as the TPP-based desorption method [34].

### 3.6. Structural Characterization

#### 3.6.1. Scanning Electron Microscope Analysis

**Figure 5:**
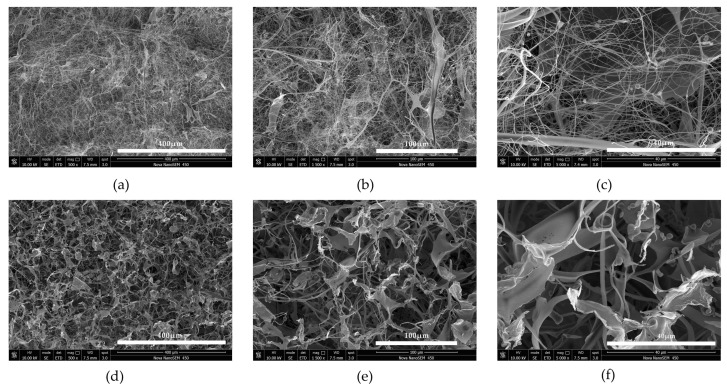
Scanning electron microscopy images of LSP-TPP at 500× (**a**), 1500× (**b**), and 5000× (**c**) and LSP-HWE at 500× (**d**), 1500× (**e**), and 5000× (**f**).

The microstructure of LSP samples extracted by different methods was observed by SEM. Figure 5 shows the morphological characteristics of 500×, 1500×, and 5000× magnification, respectively. Under 500 times magnification, LSP-HWE presented a large, irregular sheet-like agglomeration structure, with wide and thick fibers and uneven pore distribution. The overall organizational structure was loose, and the continuity was poor. LSP-TPP formed a three-dimensional network structure with uniform interlaced ultrafine fibers, no obvious large agglomeration, and the structure was regular and continuous. Under 1500 times magnification, the lamellar wrinkle morphology of LSP-HWE was clearer, but the network skeleton was rough, the regularity was poor, and the connection between fibers was loose. The LSP-TPP fibers had uniform thickness, an interwoven order, small and uniform pores, and regular and clear microscopic morphology. Under the high magnification of 5000 times, LSP-HWE was mainly composed of curled lamellae and coarse fibers, with obvious stacking and agglomeration, and the microstructure was rough and uneven. The morphology of LSP-TPP polysaccharide microfibers was straight and regular, without obvious agglomeration and visible impurities. The network was embedded with a small amount of smooth microspheres formed by self-assembly of polysaccharide chains, and the microstructure was regular and dense [35]. The above results showed that, although the traditional water extraction process could obtain LSP, it easily caused serious agglomeration of polysaccharide molecules, accompanied by residual impurities such as plant fibers. The DES-TPP three-phase extraction system had mild conditions, which not only completely retained the natural polysaccharide network structure of LSP, but also effectively alleviated molecular agglomeration and optimized the microstructure, so the structural integrity of the obtained polysaccharide was better. Combined with the results of protein content determination, LSP-TPP had no obvious aggregation of impurities such as proteins, which further confirmed that the purity of polysaccharides extracted by this method was higher.

#### 3.6.2. Relative Molecular Weight Analysis

**Figure 6:**
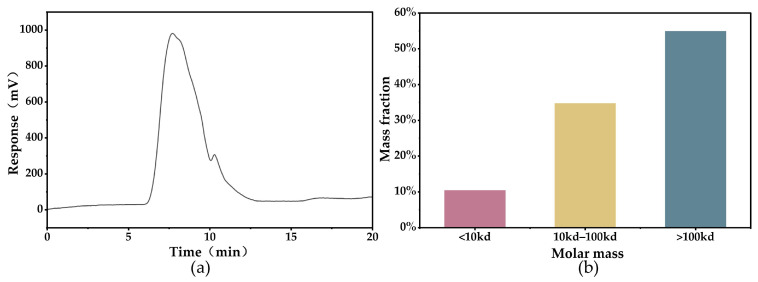
(**a**) GPC curves of LSP; (**b**) molecular weight distribution and cumulative proportion.

The molecular weight of LSP was determined by GPC with PS as the standard calibration. The GPC distribution of LSP is shown in Figure 6a. The results show that the relative molecular weight (Mw) of LSP is 375.566 kDa. It is worth noting that a small molecular fragment with a molecular weight less than 10 kDa appeared at the elution time of 10.30 min, which was also observed in the study of Yang et al. [33]. The cumulative mass fraction of LSP in different molecular weight ranges is shown in Figure 6b. It shows that LSP is mainly composed of macromolecular components. The fragments with molecular weight greater than 100 kDa accounted for a relatively high proportion, while the content of small molecular fragments (0–10 kDa) was relatively low. The above results indicated that the extraction and purification process in this experiment could effectively maintain the integrity of the polysaccharide molecular chain, and could greatly reduce the hydrolysis fracture or degradation of the polysaccharide chain, thereby ensuring stability of its biological activity.

#### 3.6.3. Fourier Transform Infrared Spectroscopic Examination

**Figure 7:**
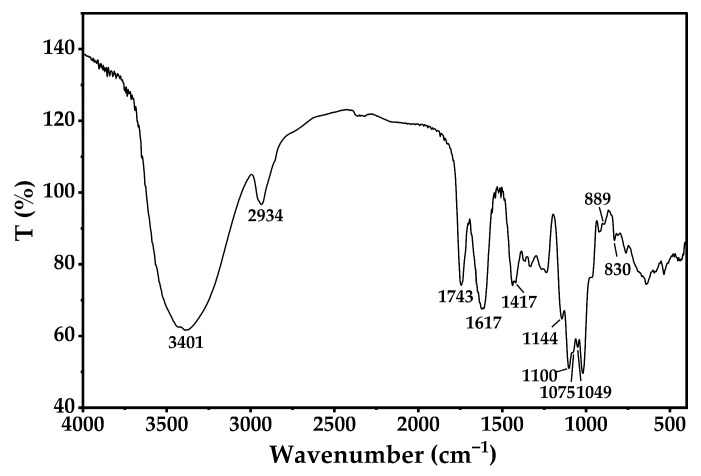
FT-IR spectrum of LSP.

The molecular structure of LSP was also studied by Fourier-transform infrared spectroscopy (FT-IR). As shown in Figure 7, the spectral patterns demonstrated were characteristic of polysaccharides. A prominent and wide absorption peak appeared at 3401 cm^−1^, which meant the stretching vibrations of the hydroxyl group. It confirmed the presence of hydrogen bonds in the polysaccharide. A weak absorption peak was detected at 2934 cm^−1^, which meant the stretching vibrations of methylene groups, which is typical of carbohydrate compounds. The simultaneous presence of the absorption peaks at 1743 cm^−1^ and 1617 cm^−1^ confirmed a pectin-like polysaccharide.

The characteristic absorption peaks observed at these wavenumbers clearly showed that we had the carbonyl stretching vibrations from esterified and free carboxyl groups. The absorption peak at 1417 cm^−1^ was identified as the symmetric stretching vibration of non-esterified carboxyl functional groups. In the fingerprint region of 1000 to 1200 cm^−1^, there were multiple well-defined absorption bands at 1144, 1100, 1075 and 1049 cm^−1^, which were attributed to the vibrations of the glycosidic C-O-C bonds and hydroxylated carbon atom. The most intense peak at 1049 cm^−1^ mainly came from C-O stretching vibrations that occurred in the furanose ring configuration. The absorption bands that appear at 1100 and 1075 cm^−1^ correspond to the structural elements of pyranose units, especially that of galactose and glucose moieties. In the spectral range that gives the conformations of the glycosidic linkage, the absorption band near 830 cm^−1^ was a mark for the a-configuration glycosidic bonds, as it demonstrates its structural properties. As can be seen in [14], the main framework of pectin is made of the residues of alpha-D-galacturonic acid [36]. The weak signal at 889 cm^−1^ suggests the presence of bonds of the beta type, which can come from galactose or xylose parts of the branching structures of the polysaccharide.

#### 3.6.4. Monosaccharide Composition Analysis

**Figure 8:**
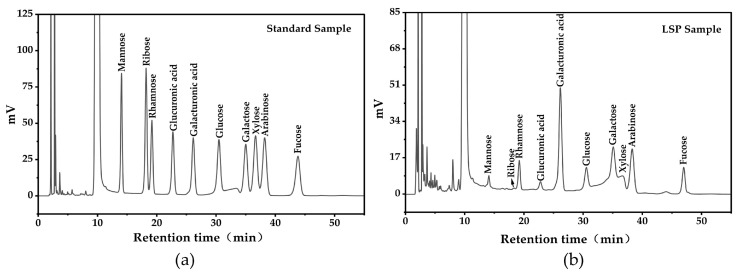
The monosaccharide composition of the standard sample (**a**) and the LSP sample (**b**).

Studies have shown that the biological activity of polysaccharides, such as antioxidant capacity, is related to the monosaccharide composition of polysaccharides [37,38]. PMP derivatization HPLC analysis was used to measure the content of monosaccharides. The analysis results are shown in Figure 8 and Table 6. Figure 8a and Figure 8b show the chromatograms of the standard monosaccharide mixed solution and the LSP sample solution, respectively. Table 6 shows the results of the component calculation and analysis of LSP. An LSP chromatogram showed that LSP was mainly composed of galacturonic acid, arabinose, and galactose, and contained a certain amount of rhamnose, glucose, glucuronic acid, xylose, and mannose, as well as trace amounts of fucose and ribose. The contents of these monosaccharides were 29.550 mg/g, 11.137 mg/g, 10.191 mg/g, 6.654 mg/g, 6.227 mg/g, 2.290 mg/g, 2.092 mg/g, 1.933 mg/g, 0.998 mg/g and 0.214 mg/g, respectively. The molar ratio of these monosaccharides calculated from the peak area was 37.86:18.45:14.07:10.08:8.60:3.47:2.93:2.67:1.51:0.35, so the major monosaccharides in LSP are galacturonic acid, arabinose, and galactose. The presence of galacturonic acid indicated that LSP is an acidic heteropolysaccharide [39].

### 3.7. Physicochemical Properties of LSP

#### 3.7.1. pH

The pH value of polysaccharides is a very important physical and chemical property, which determines the chemical conformation, physical properties of polysaccharides, and the performance of polysaccharides in complex systems to some extent [40,41,42]. As shown in Table 5, the pH value of LSP-HWE was 5.07 ± 0.03, and the pH value of LSP-DES was 4.18 ± 0.02, indicating that LSP is an acidic heteropolysaccharide. It is worth noting that the pH value of LSP-DES was lower than that of LSP-HWE. This difference may be related to the specific enrichment of acidic polysaccharide components by DES extraction. Studies have shown that the polysaccharide extracted by acidic DES contains more glucuronic acid, which was confirmed by the studies of Wang et al. and Zhang et al. [42,43].

#### 3.7.2. Solubility

Solubility is an important index to evaluate the physicochemical properties of polysaccharides, which directly affects their application potential in food, medicine, and other fields [44]. The water solubility of LSP-HWE and LSP-TPP at 25 °C, 60 °C, and 80 °C was determined. The results in Table 7 show that the solubility of the two polysaccharide samples increased significantly with increasing temperature. This phenomenon is related to the physical and chemical mechanisms of polysaccharide dissolution. Increasing temperature will promote more intense molecular movement in the water environment. This intensified thermal motion weakens the extensive intermolecular hydrogen bond network that normally stabilizes the aggregated polysaccharide chain and induces conformational rearrangement, exposing more hydrophilic groups to the solvent. The net effect is that the aggregation tendency is reduced, and the overall solubilization efficiency is significantly improved [45,46]. Sharifian-Nejad et al. also observed a similar rule in the study of polysaccharides from Elaeagnus angustifolia fruit, and found that the solubility of polysaccharides increased with the increase in temperature, which was attributed to the effect of heat treatment on the molecular structure of polysaccharides [47]. At the same time, it could be noted that the solubility of LSP-DES was higher than that of LSP-HWE at the same temperature, indicating that DES extraction can significantly improve the water solubility of longan shell polysaccharides. Muhammad Hasnun Md Yusoff et al. pointed out that a wide range of hydrogen bond networks were formed between DES and hydrophilic polar polysaccharide groups, which enhanced their hydrophilicity and increased the solubility of polysaccharides [8].

#### 3.7.3. Color Profile

The CIE Lab color difference analysis results are shown in Table 5. The total color difference ΔE between the polysaccharide solution extracted by LSP-TPP and the ultrapure water standard sample was only 0.69, and the solution was highly clear and transparent. The ΔE of the polysaccharide solution extracted by LSP-HWE reached 2.08, showing an obvious dark reddish color. This result fully confirmed that, compared with the traditional water extraction process, DES extraction technology could effectively avoid side reactions such as Maillard browning and pigment co-extraction in the polysaccharide extraction process through mild extraction conditions. The introduction of colored impurities was greatly reduced, and finally, a polysaccharide product with higher purity and better color was obtained, which provided quality assurance for the high-value application of polysaccharides.

#### 3.7.4. Zeta Potential

The measurement of zeta potential gives some information about the character of the stability of the colloidal systems [48]. In this study, the electrical charge properties of LSP-HWE and LSP-TPP at different pH levels were studied and the data are shown in Table 5. As can be seen, at all the tested pH levels (3.0–11.0), both of the LSPs have negative surface charges, which confirms that they are the negatively charged polysaccharides [49]. The classification of the colloidal systems is generally done by their zeta potential (ZP) measurements: the dispersions with a ZP value in the range (in module) of 0 to 10 mV are classified as very unstable, values (in module) from 10 to 20 mV show some stability, systems with ZP (in module) from 20 to 30 mV have an intermediate stability, and when the absolute ZP (in module) is above 30 mV the dispersion is considered very stable [50]. In the case of the LSP-DES formulation, excellent colloidal stability was observed, as the ZP measurements were always above 30 mV in the pH spectrum of 5.0–11.0, indicating a very low tendency for particle aggregation or sedimentation. In the case of the very low pH (pH = 3.0) for both LSP specimens, the ZP values are below 20 mV, which corresponds to the diminished colloidal stability. From the analysis of the zeta potential values, it was found that LSP-TPP always showed bigger absolute values than LSP-HWE in the whole range of pH, probably because there is a higher concentration of glucuronic acid in LSP-TPP [51]. Also, these results have been corroborated by the results of the pH measurement. The extraction methodology that we implemented in this work is better than the usual hot water extraction in that it has polysaccharides with much bigger zeta potential values, which improves the stability of the colloidal systems. These kinds of properties make the LSP-TPP suitable for different applications in both the pharmaceutical and food sectors [52,53].

### 3.8. Functional Properties

#### 3.8.1. Thermal Analysis

**Figure 9:**
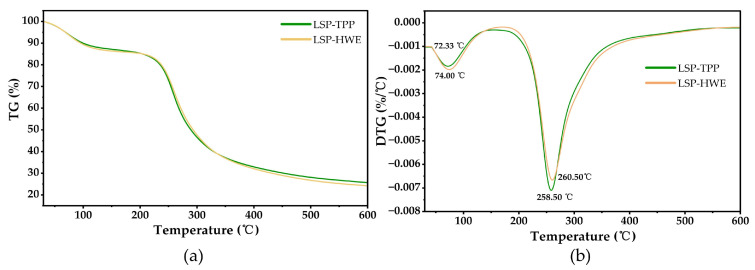
The TG (**a**) and DTG (**b**) curves of LSP.

The thermal properties of LSP were analyzed by thermogravimetry (TG) and derivative thermogravimetric (DTG) curves (Figure 9). The results of synergistic analysis showed that the pyrolysis behavior of longan shell polysaccharides extracted by LSP-TPP and LSP-HWE was highly consistent, showing a typical three-stage pyrolysis mode. At the macro level, the downward trend of the two TG curves was almost coincidental, indicating that the pyrolysis rate of the two polysaccharides was highly consistent with the mass loss model. However, the residual carbon content of LSP-TPP at 600 °C (25.64%) was significantly higher than that of LSP-HWE (24.22%), reflecting the stronger carbonization ability of LSP-TPP. At the micro level, both DTG curves showed two characteristic peaks, and the low temperature peak (72.33 °C vs. 74.00 °C) corresponded to the volatilization of bound water and small molecular impurities. The lower weight loss rate of LSP-TPP (13.00% vs. 13.41%) suggested a slightly lower bound water content. But we did not test if this difference is statistically significant. The high temperature peak (258.50 °C vs. 260.50 °C) corresponded to the cleavage and degradation of the main chain glycosidic bond of the polysaccharide. The slightly wider peak shape of LSP-TPP reflected that its pyrolysis process was more complex and may contain the synergistic degradation of multiple polysaccharide components. According to the results of monosaccharide composition analysis and infrared spectroscopy, DES extraction not only completely retained the natural pyrolysis characteristics of longan shell polysaccharides but also selectively enriched acidic sugar components such as galacturonic acid. These acidic groups significantly enhanced the carbonization ability of polysaccharides by forming intramolecular hydrogen bonds, so that LSP-TPP showed better thermal stability than LSP-HWE, which provided a solid scientific basis for DES as a green extraction solvent to replace the traditional water extraction method and promote the high-value application of longan shell polysaccharides in the fields of food and medicine.

#### 3.8.2. Antioxidant Potential

**Figure 10:**
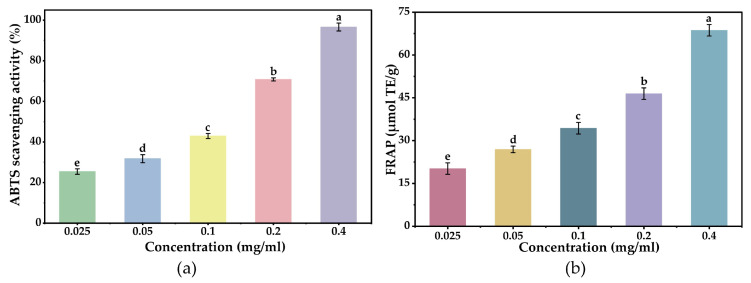
Variation in ABTS radical scavenging percentage with varying concentrations for LSP (**a**), the antioxidant capacity of FRAP (**b**). The data were calculated from three parallel experiments. Different letters indicate statistical significance at *p* < 0.05.

LSP showed significant concentration-dependent antioxidant activity in ABTS free radical scavenging and FRAP reducing power tests (Figure 10). The ABTS radical scavenging activity of LSP-TPP increased with concentration and reached 96.6 ± 2.0% at 0.4 mg/mL. The EC50 came out around 0.13 mg/mL. For comparison, Yang et al. [33] reported an EC50 of 0.21 mg/mL for hot water-extracted longan shell polysaccharides under similar conditions. The lower EC50 of LSP-TPP suggests the DES-TPP method does not harm the radical scavenging capacity. Several observations point to possible mechanisms. The high galacturonic acid content of LSP-TPP (37.86%) is a likely factor. Carboxyl groups from galacturonic acid can donate electrons and chelate metal ions, both mechanisms known to help neutralize radicals. Some studies have used chemical modification to test this directly. Chen et al. [37] found that when the carboxyl groups of pectic polysaccharides are esterified, ABTS scavenging activity drops substantially. Our data cannot prove causality, but the correlation is consistent with that finding. Another factor is morphology. The SEM images showed LSP-TPP forming an open, fibrous network, while LSP-HWE appeared as dense aggregates. Similar observations have been made for DES-extracted polysaccharides from other sources [44]. The proposed explanation is that a looser structure improves the accessibility of reactive groups to radicals in solution. Our solubility data support this idea: LSP-TPP dissolved almost completely at 80 °C (98.5%), compared to 95.1% for LSP-HWE. Better solubility likely means more of the polysaccharide is actually available to react.

The FRAP assay showed a similar concentration-dependent trend. The ferric-reducing power reached 68.67 ± 2.02 μmol TE/g at 0.4 mg/mL. The FRAP value of LSP-TPP appears proportionally higher than its ABTS activity when compared to LSP-HWE. This might reflect a higher density of reducing ends, but we cannot be certain without more detailed structural data. We need to acknowledge a few limitations. The EC50 came from a limited concentration range (0.025–0.4 mg/mL). A wider range would give a more reliable estimate. More importantly, our interpretation that galacturonic acid drives the antioxidant activity is indirect at this stage. To prove causality, one would need to chemically modify the carboxyl groups—for example, by esterification—and test whether activity drops. Molecular weight may also matter. Some studies have found that polysaccharides with lower molecular weight show better antioxidant activity [54]. LSP-TPP has a molecular weight of 375.6 kDa, which is within the 10–1000 kDa range that is widely recognized for remarkable bioactivity [3]. Whether the DES-TPP method changes molecular weight distribution compared to hot water extraction is worth exploring in future work.

Despite these caveats, the data clearly show that LSP-TPP has strong in vitro antioxidant activity. The DES-TPP extraction method did not compromise this bioactivity; in fact, LSP-TPP performed as well as or better than conventionally extracted polysaccharides. This supports the potential use of LSP as a natural antioxidant in food or nutraceutical applications.

## 4. Discussion

It should be noted that the comparison between DES-TPP and tert-butanol TPP extracts was limited to extraction yield; structural and antioxidant comparisons were not performed. As the primary objective was to develop a greener alternative, this limitation does not affect the main conclusions. Nevertheless, the higher yield, comparable purity, and the non-toxic, recyclable nature of DES already demonstrate its advantages over tert-butanol for food applications. Future studies should include a direct comparative characterization to fully confirm equivalence.

Despite the above conclusion, this study still has some limitations. First, no antibacterial experiment was carried out in this study. Studies have shown that many plant polysaccharides have both antioxidant and antibacterial activities, and the lack of antibacterial experiments limits our exploration of the potential multifunctional biological activity of the polysaccharide. Second, all the data in this study were from in vitro experiments, and no in vivo experiments were carried out. Therefore, the absorption, metabolism, and bioavailability of LSP in vivo and its protective effect on oxidative stress are still unclear. To make up for these limitations, future research should include the following contents: (1) Systematic antibacterial experiments should be carried out to determine the minimum inhibitory concentration of LSP and other data, and to explore the potential relationship between antioxidant and antibacterial activity. (2) Animal experiments should be done to check oxidative stress biomarkers and carry out histopathological examination. (3) Structure–activity relationship studies should be performed to try to correlate the structure of molecular weight, glycosidic bond connection, chain conformation, and branching degree with antioxidant activity. There should be further focus on the evaluation of antioxidant, anti-tumor, and other biological activities of LSP-DES, and further exploration of its metabolism and mechanism of action in vivo, to lay a theoretical foundation for its application in functional food or medicine.

## 5. Conclusions

In this study, an integrated and sustainable strategy was established to combine ultrasound with a DES-mediated three-phase distribution system for the separation of polysaccharides from longan shell biomass. The hydrophobic eutectic mixture derived from dodecanoic acid and octanoic acid (molar ratio 1:1) as a phase separation agent represents a greener and safer alternative to the conventionally used tert-butanol. Through a single-factor test combined with the response surface method, the optimum extraction process parameters were determined as follows: liquid-to-solid ratio 1:20 g/mL, upper-to-lower phase volume ratio 1:1.04 *v*/*v*, ammonium sulfate concentration 26.3 wt%, ultrasonic time 25 min, and extraction temperature 63.8 °C. Under the optimized conditions, the polysaccharide yield could reach 2.42 ± 0.03%. At the same time, the deep eutectic solvent showed excellent reusability. After five cycles of extraction, the yield dropped from 2.42% to about 1.92%—a decrease of about 0.5 percentage points, retaining approximately 80% of the original yield. The main monosaccharide composition of LSP was galacturonic acid and arabinose. The characterization results of physicochemical properties showed that the polysaccharide extracted by the UA-TPP-DES process, LSP-TPP, had better physicochemical properties than LSP-HWE. Functional activity evaluation confirmed that LSP had favorable thermal stability and outstanding antioxidant activity. At a concentration of 0.4 mg/mL, the ABTS free radical scavenging rate was as high as 96.6 ± 2.0%, and the FRAP antioxidant capacity was 68.67 ± 2.02 μmol Trolox/g. Future work should include in vivo antioxidant tests and structure–activity studies to better understand how it works. The process developed in this study not only significantly improved the extraction efficiency and product quality of LSP but also provided a sustainable and promising technical path for the large-scale green production of LSP and the development of functional foods. Furthermore, the UA-DES-TPP method uses recyclable DES and mild conditions, which may offer economic benefits for large-scale applications. This approach could also be extended to extract bioactive polysaccharides from other agricultural byproducts.

## Figures and Tables

**Table 1:** Different compositions of DESs.

NO.	Solvent Composition	Molar Ratio
DES-1	Lauric acid:octanoic acid	1:1
DES-2	Lauric acid:nonanoic acid	1:1
DES-3	Lauric acid:decanoic acid	1:1

**Table 2:** Factors and levels for Box–Behnken center combination experimental design.

Levels	Factors		
	A	B	C
	Extraction Temperature (°C)	Mass Fraction of (NH_4_)_2_SO_4_ (wt%)	Volume Ratio of the Top Phase to the Bottom Phase (*v*/*v*)
−1	55	0.20	0.5:1
0	65	0.25	1:1
1	75	0.30	1.5:1

**Table 3:** Box–Behnken experimental design with the actual and response values for the extraction yield.

Run	Extraction Temperature (°C)	Mass Fraction of (NH_4_)_2_SO_4_ (wt%)	The Volume Ratio of the Upper and Lower Phases (*v*/*v*)	Yield (%)
1	55	30	1	2.11 ± 0.03
2	65	25	1	2.41 ± 0.04
3	65	25	1	2.41 ± 0.05
4	65	30	0.5	2.00 ± 0.02
5	75	25	1.5	1.91 ± 0.03
6	75	20	1	1.84 ± 0.05
7	55	25	0.5	2.03 ± 0.04
8	65	25	1	2.37 ± 0.04
9	55	25	1.5	1.66 ± 0.03
10	65	25	1	2.47 ± 0.07
11	65	20	1.5	1.59 ± 0.05
12	75	25	0.5	1.63 ± 0.04
13	75	30	1	1.93 ± 0.03
14	65	20	0.5	1.79 ± 0.04
15	65	25	1	2.42 ± 0.05
16	55	20	1	1.79 ± 0.02
17	65	30	1.5	1.94 ± 0.07

**Table 4:** Regression model analysis of variance.

Source	Sum of Squares	d*f*	Mean Square	F-Value	*p*-Value	Significant
Model	1.43	9	0.1590	99.39	<0.0001	***
A	0.0105	1	0.0105	6.57	0.0373	*
B	0.1201	1	0.1201	75.06	<0.0001	***
C	0.0153	1	0.0153	9.57	0.0175	*
AB	0.0121	1	0.0121	7.57	0.0285	*
AC	0.1056	1	0.1056	66.05	<0.0001	***
BC	0.0064	1	0.0064	4.00	0.0856	n.s.
A^2^	0.2830	1	0.2830	176.95	<0.0001	***
B^2^	0.2461	1	0.2461	153.87	<0.0001	***
C^2^	0.5136	1	0.5136	321.13	<0.0001	***
Residual	0.0110	7	0.0016			
Lack of fit	0.0060	3	0.0020	1.60	0.3127	n.s.
Pure Error	0.0050	4	0.0012			
Cor Total	1.43	16				
R^2^	0.9923					
Adjusted R^2^	0.9825					
Predicted R^2^	0.9275					

* Statistical significance (*p* ≤ 0.05); high significance (*p* ≤ 0.01); *** extreme significance (*p* ≤ 0.001); n.s., no statistical significance (*p* > 0.05).

**Table 5:** The yield, sugar content, and protein content of LSP prepared through UA-DES-TPP extraction.

	Test Value (%)	RSD (*n* = 3)
LSP extraction yield	2.42 ± 0.03	1.04%
Sugar	89.03 ± 0.43	0.48%
Protein	1.83 ± 0.07	3.55%

**Table 6:** Analysis of monosaccharide composition of the sample solution.

Monosaccharide Composition	Retention Time (min)	Content (mg/g)	Molar Ratio (%)
Mannose	4.079	1.933	2.67
Ribose	0.451	0.214	0.35
Rhamnose	14.041	6.654	10.08
Glucuronic acid	4.832	2.290	2.93
Galacturonic acid	62.351	29.550	37.86
Glucose	13.139	6.227	8.60
Galactose	21.502	10.191	14.07
Xylose	4.415	2.092	3.47
Arabinose	23.500	11.137	18.45
Fucose	2.105	0.998	1.51

**Table 7:** The pH, solubility, color properties, and zeta potential of LSP extracted by TPP and HWE.

	LSP-TPP	LSP-HWE
**pH**	4.18 ± 0.02 ^b^	5.07 ± 0.03 ^a^
**Solubility (%)**		
**25 °C**	91.87 ± 1.10 ^a^	86.40 ± 0.82 ^b^
**60 °C**	94.53 ± 1.00 ^a^	91.97 ± 1.07 ^a^
**80 °C**	98.50 ± 1.06 ^a^	95.10 ± 0.87 ^b^
**Color properties**		
L*	27.01 ± 0.08 ^a^	24.49 ± 0.05 ^b^
a*	2.23 ± 0.06 ^b^	3.32 ± 0.02 ^a^
b*	3.31 ± 0.02 ^b^	3.48 ± 0.01 ^a^
**Zeta potential (mV)**		
**pH = 3**	−15.33 ± 1.16 ^a^	−7.04 ± 0.17 ^b^
**pH = 5**	−23.70 ± 1.04 ^a^	−8.46 ± 0.21 ^b^
**pH = 7**	−26.90 ± 0.44 ^a^	−12.47 ± 0.35 ^b^
**pH = 9**	−32.10 ± 0.61 ^a^	−15.73 ± 0.15 ^b^
**pH = 11**	−37.93 ± 0.15 ^a^	−29.03 ± 0.81 ^b^

Different letters indicate statistical significance at *p* < 0.05, *n* = 3.

## Data Availability

The original contributions presented in this study are included in the article. Further inquiries can be directed to the corresponding author.
